# MMonitor for real-time monitoring of microbial communities using long reads

**DOI:** 10.1016/j.crmeth.2025.101266

**Published:** 2025-12-23

**Authors:** Timo N. Lucas, Ulrike Biehain, Anupam Gautam, Kurt Gemeinhardt, Tobias Lass, Simon Konzalla, Ruth E. Ley, Largus T. Angenent, Daniel H. Huson

**Affiliations:** 1 Institute for Biomedical Informatics (IBMI), University of Tübingen, Sand 14, 72076 Tübingen, Germany; 2 Cluster of Excellence “Controlling Microbes to Fight Infections” (CMFI), University of Tübingen, Auf der Morgenstelle 28, 72074 Tübingen, Germany; 3 Environmental Biotechnology Group, Department of Geosciences, University of Tübingen, Schnarrenbergstraße 94–96, 72076 Tübingen, Germany; 4 Department of Biological and Chemical Engineering, Aarhus University, Gustav Wieds Vej 10D, 8000 Aarhus C, Denmark; 5 The Novo Nordisk Foundation CO_2_ Research Center (CORC), Aarhus University, Gustav Wieds Vej 10C, 8000 Aarhus C, Denmark; 6 Ag Angenent, Max Planck Institute for Biology, 72076 Tübingen, Germany; 7 Department of Microbiome Science, Max Planck Institute for Biology Tübingen, 72076 Tübingen, Germany

**Keywords:** real-time metagenomics, nanopore sequencing, bioinformatics software, microbiome, data visualization, biotechnology

## Abstract

Real-time monitoring of microbial communities offers valuable insights into microbial dynamics across diverse environments. However, many existing metagenome analysis tools require advanced computational expertise and are not designed for monitoring. We present MMonitor, an open-source software platform for real-time analysis and visualization of metagenomic Oxford Nanopore Technologies (ONT) sequencing data. MMonitor includes two components: a desktop application for running bioinformatics pipelines through a graphical user interface (GUI) or command-line interface (CLI) and a web-based dashboard for interactive result inspection. The dashboard provides taxonomic composition over time, quality scores, diversity indices, and taxonomy-metadata correlations. Integrated pipelines enable automated *de novo* assembly and reconstruction of metagenome-assembled genomes (MAGs). To validate MMonitor, we tracked human gut microbial populations in three bioreactors using 16S rRNA gene sequencing and applied it to whole-genome sequencing (WGS) data to generate high-quality annotated MAGs. We compare MMonitor with other real-time metagenomic tools, outlining their strengths and limitations.

## Highlights

• Real-time monitoring of microbial communities from nanopore sequencing
• Taxonomic, functional, and time-series analyses with automated database updates
• Enables high-quality assembly and annotation of metagenome-assembled genomes
• Open-source platform combining GUI, CLI, and web dashboard for accessible tracking

## Motivation

Metagenome monitoring is essential for understanding and managing microbial communities in dynamic environments. However, existing tools for metagenome analysis often lack key features needed for effective real-time monitoring—including immediate processing of nanopore data, intuitive user interfaces, simple deployment, and clear visual summaries. Many workflows require advanced computational skills, manual integration of multiple software packages, or substantial hardware resources, which can delay responses in biologically active systems such as clinical diagnostics, industrial bioprocesses, and environmental surveillance.

MMonitor addresses these limitations by providing real-time analysis of nanopore sequencing data within an accessible, easy-to-configure platform.

## Graphical abstract

**Figure:**
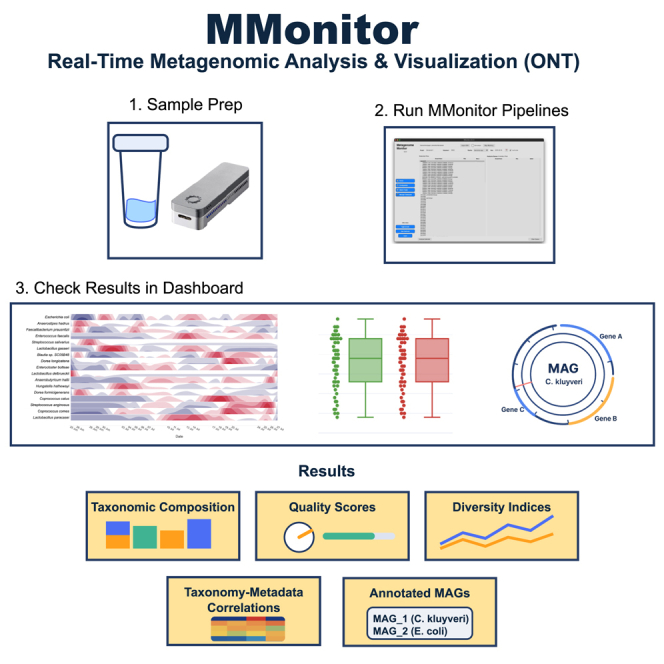

## Teaser

Lucas et al. present MMonitor, an open-source software enabling real-time monitoring of microbial communities using nanopore sequencing. The tool integrates taxonomic and functional analyses with automated visualization and metagenome assembly, allowing researchers to track microbiome dynamics and identify community changes during ongoing sequencing runs.

## Introduction

### Monitoring metagenomes through long-read sequencing

Metagenomics has advanced our understanding of microbial diversity and function in diverse environments.^1^^,^^2^ Sequencing and bioinformatic analysis are applied to bioactive systems such as anaerobic digesters, where they support process optimization, or human microbiomes, where they provide insights into host-microbiome interactions and health.^2^^,^^3^^,^^4^ Metagenomic monitoring also links microbial dynamics to biogeochemical processes in larger ecosystems such as oceans and soils.^5^^,^^6^

Traditional assays (optical, fluorescence based, or qPCR) sensitively quantify specific taxa but are limited to predefined targets and cannot capture overall community dynamics. Long-read sequencing technologies, such as ONT platforms, overcome these limitations. Compared to short-read sequencing, long reads provide higher taxonomic resolution and simplify the assembly of complete genomes from metagenomes or isolates, without requiring complementary short-read data.^7^^,^^8^ High-quality genomes are essential for detecting structural variations^9^ and improving functional annotations.^10^

Analyzing long-read data poses unique computational challenges. Algorithms developed for short reads often struggle with higher error rates and longer sequence lengths. Efficient methods are essential for real-time monitoring, particularly in time-sensitive contexts such as pathogen detection, where rapid analysis can aid in interventions. Several algorithms for long-read analysis have emerged: MetaMaps^11^ and Emu^12^ were designed specifically for this purpose, while Centrifuge^13^ and Kraken2,^14^ though originally developed for short reads, have also proven useful for long-read metagenomics.^15^

Nanopore sequencing has already been applied to diverse real-time monitoring tasks, including tracking resistance genes,^16^ identifying outbreaks,^17^ and pathogen surveillance in insects^18^ and freshwater systems.^19^ Real-time analysis is particularly valuable in industrial settings, where undetected contamination can cause costly production losses. The Oxford Nanopore Technologies (ONT) MinION, with its portability and rapid turnaround, has even been deployed outside conventional laboratories, including on the International Space Station^20^ and on ocean research vessels.^21^

Several tools already implement real-time monitoring, including minoTour,^22^ NanopoReaTA,^23^ MARTi,^24^ and Nanometa Live,^25^ but they face challenges in scalability, ease of use, and flexibility. Many focus heavily on taxonomic profiling without a real-time component, while others lack features such as time-series visualization or effective sample management. More comprehensive pipelines, such as HUMAnN^26^ or QIIME,^27^ can generate detailed reports but often require command-line expertise, limiting accessibility. Online platforms such as MG-RAST,^28^ BusyBee,^29^ and BugSeq^30^ provide user-friendly interfaces, but do not process continuous sequencing streams. Without frequent database updates, sensitivity also declines due to outdated references.

With these limitations in mind, we developed MMonitor, a software platform optimized for real-time microbial monitoring based on ONT long-read sequencing.

### Design

We designed MMonitor considering the following:

• Intuitive user interfaces (graphical and command line) that appeal to researchers of varying levels of computational skill.
• Ability for time-series monitoring to track taxonomic shifts as new reads arrive.
• Customizable pipelines for 16S rRNA gene and whole-genome sequencing (WGS)-based taxonomic and functional analysis including metagenome assembly.
• Up-to-date databases with the ability to update automatically.
• Easy operation and installation.
• Useable on high-end consumer-grade hardware.
• Remote data access from any device or local processing.

A comparative analysis of our method with other tools in the real-time metagenomics space (e.g., minoTour,^22^ NanopoReaTA,^23^ MARTi,^24^ Nanometa Live,^25^ MAIRA,^31^ EPI2ME,^32^ BoardION,^33^ and CRuMPIT^34^) can be found in the results section.

## Results

### Metagenome monitoring software

Here, we illustrate the use of MMonitor, an open-source software for automated monitoring through the real-time analysis of metagenomic nanopore sequencing data. The software consists of two components: a desktop application for running analyses and a web-based application for interactive inspection of results. The desktop part integrates established bioinformatics pipelines that can be run through a graphical user interface (GUI) or a command-line interface (CLI), enabling analysis on local or remote systems. The software leverages the real-time characteristics of nanopore data, allowing automated processing of metagenome reads as the sequencer continuously generates data.

The web application features a visualization dashboard that provides dynamic insights into taxonomic composition over time and the functional potential of metagenomes. It includes key metrics such as quality scores, diversity indices, and taxonomy-metadata correlations, with options to export results for further analysis. MMonitor was developed and tested in collaboration with environmental biotechnologists to ensure its applicability for on-site metagenome tracking.

In the following sections, we describe how the software was used to track microbial communities in multiple bioreactors over a month using 16S rRNA gene sequencing data. We then illustrate how MMonitor processes WGS data to generate annotated, high-quality metagenome-assembled genomes (MAGs) for functional insights. Finally, we compare MMonitor with other existing tools for metagenome monitoring.

**Figure 1:**
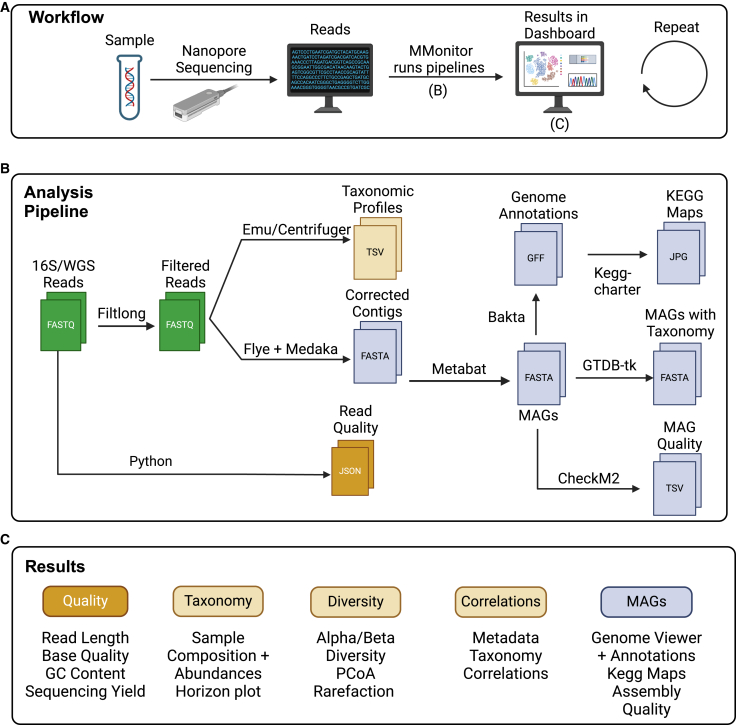
Overview of the MMonitor workflow and outputs (A) Typical laboratory workflow using MMonitor for real-time metagenome tracking. (B) Computational steps in the taxonomic and functional pipelines. (C) Examples of dashboard outputs (time-series taxonomy, QC, diversity, and correlations). Created with biorender.com.

Data sequenced at regular intervals (e.g., weekly samples in our study, although the frequency depends on the application) are automatically analyzed by MMonitor Desktop, and results are sent to the dashboard. Data can be accessed locally or through a web browser. MMonitor offers two pipelines (Figure 1B): one for taxonomic analysis and one for functional analysis. The taxonomic pipeline processes both 16S rRNA gene sequencing and WGS reads, whereas the functional pipeline requires WGS data.

16S rRNA gene sequencing provides rapid taxonomic profiles, and multiplexing allows simultaneous analysis of multiple samples. In contrast, WGS offers deeper taxonomic resolution and strain-level insights but requires more time and computational resources. WGS also enables *de novo* assembly for full genome insights. We recommend 16S rRNA gene sequencing for general community overviews and WGS at key intervals for in-depth analysis. Details of the pipelines are provided in STAR Methods.

### 16S rRNA gene time series in three BES reactors

This section reports nanopore 16S rRNA gene time series data from three bioelectrochemical systems (BESR1–BESR3). In total, 66 amplicon libraries collected between July and August 2023 were analyzed with MMonitor. For clarity, BESR1–BESR3 are hereafter referred to simply as R1–R3. Unless noted otherwise, all mentions of R1–R3 refer exclusively to the 16S datasets from these BES reactors. Genome-resolved WGS results from a separate reactor are described in a later section and are based on a single sample.

The bioelectrochemical systems originated from a parallel project designed to test whether removing H_2_ with electrodes could steer anaerobic fermentation pathways. The reactors achieved stable H_2_ removal and produced a 16S rRNA gene sequencing time series. We primarily used these data to validate MMonitor: frequent sampling under a defined perturbation provided an excellent test case for real-time tracking, horizon plots, and diversity analyses.

Reads were quality-filtered using the 16S defaults (removing reads <1,000 bp, >2,000 bp, or with PHRED <10). The remaining reads were used for downstream taxonomic profiling and diversity analyses.

**Figure 2:**
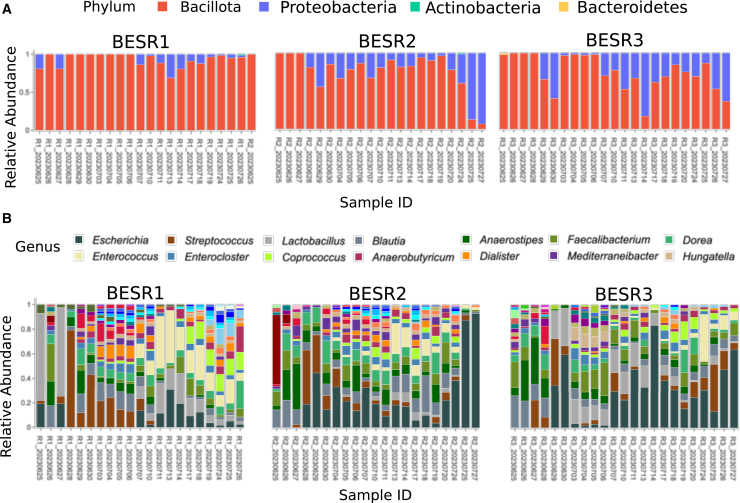
Community composition across three BES reactors Legends display only the 14 most abundant taxa. (A) Stacked bar plots of relative abundance at the phylum level for R1–R3. (B) Stacked bar plots at the genus level for R1–R3. Created with biorender.com.

Here, we analyze the taxonomy of the three monitored bioreactors at different taxonomic ranks (Figure 2). Across the three BES reactors (R1, R2, and R3), microbial communities were consistently dominated by the phylum Bacillota, with smaller contributions from Proteobacteria, Actinobacteria, and Bacteroidetes. Over the course of monitoring, MMonitor showed that community composition (Figure 2A) was relatively stable at higher taxonomic ranks, with Bacillota dominating throughout. At finer resolutions, fluctuations became more apparent.

At the genus level, taxa such as *Escherichia*, *Streptococcus*, *Lactobacillus*, *Blautia*, *Anaerostipes*, and *Faecalibacterium* dominated alongside other minor genera (Figure 2B). In summary, periodic variations were observed at finer taxonomic ranks, while higher ranks showed a more stable equilibrium dominated by Bacillota and Proteobacteria.

**Figure 3:**
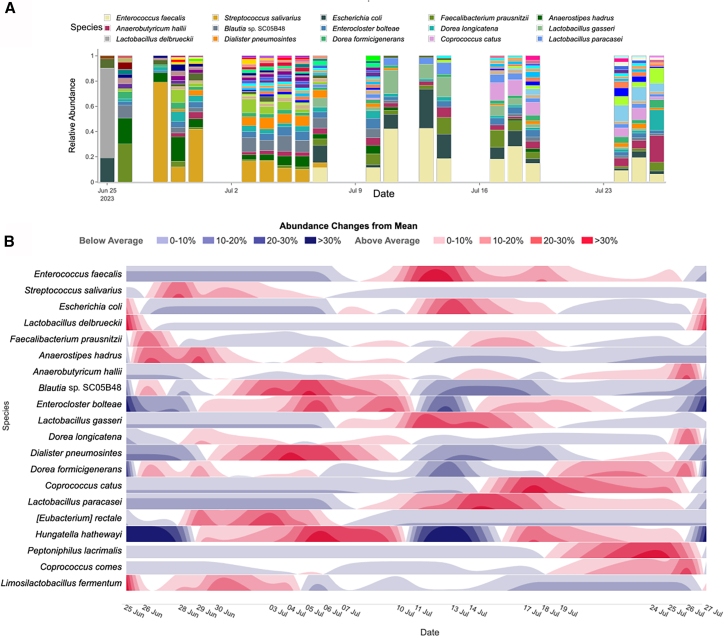
Species-level dynamics in reactor R1 (A) Stacked bar plot of the most abundant species over time. (B) Horizon plot showing deviations from mean abundance for the 20 most abundant species (red, above mean; blue, below mean). Created with biorender.com.

In reactor R1, the five most abundant species were *Enterococcus faecalis*, *Streptococcus salivarius*, *Escherichia coli*, *Faecalibacterium prausnitzii*, and *Anaerostipes hadrus*. Species-level changes were more pronounced: while daily variation was often minor, over the course of weeks the community was unstable, with some taxa emerging and others disappearing (Figure 3A).

These dynamics are visualized in a horizon plot (Figure 3B), which shows relative abundance changes of the 20 most abundant species in R1 over time. Each row represents a species: red bands indicate increases compared to the mean abundance and blue bands indicate decreases. For example, *S. salivarius* and *Dorea longicatena* increased early, then declined, while *E. coli* and *E. faecalis* only appeared after July 10, resulting in continuous blue bands before that date followed by red increases afterward. Such patterns highlight potential key time points, e.g., a marked community shift around July 11 or earlier changes on June 25 affecting taxa such as *Lactobacillus delbrueckii*, *F. prausnitzii*, and *A. hadrus*.

Community shifts in the BES reactors were also described in detail in a previous dissertation based on the same dataset.^35^ That study reported marked changes at 201, 354, and 509 h, with differences between replicates and two enrichment phases in R1 (0–226 h and 251–559 h). These shifts were associated with metabolite concentrations (e.g., acetate, propionate, *n*-valerate) and biofilm growth at electrodes, which likely influenced hydrogen availability. The exact causes of the observed species turnovers remain unresolved. As the goal of the present study was to demonstrate MMonitor’s ability to capture such dynamics in real time, we focused on showcasing monitoring capabilities rather than providing a full biological interpretation.

### Analysis based on *de novo*-assembled MAGs and functional analysis

To demonstrate additional use cases of our software, we also applied it to WGS data. This is particularly important, as 16S rRNA gene sequences alone are often unable to differentiate between functionally distinct microbial taxa. Closely related species or strains with nearly identical 16S rRNA genes may possess different genomic content and functional capabilities.^36^ WGS data also enable the tracking of other phylogenetic and functional marker genes beyond 16S rRNA, which is particularly valuable in complex biological environments where distinct taxa may fulfill similar ecological roles.

MMonitor successfully generated MAGs and provided insight into the functional potential of individual microbes. From a nanopore WGS dataset, we obtained seven high-quality MAGs, which met the Minimum Information about a Metagenome-Assembled Genome (MIMAG) standards^37^ as assessed by CheckM2.^38^

Taxonomic annotation using GTDB-Tk (Genome Taxonomy Database) revealed that six MAGs belonged to bacteria and one to Archaea (Table 1). Two MAGs were classified down to the species level: *Pseudoclavibacter_A caeni* and *Methanobacterium_C congolense*, with ANI (average nucleotide identity) values of 99.28% and 97.88%, respectively, indicating high sequence similarity to known reference genomes. The remaining MAGs were classified at the genus level, including *Dysosmobacter*, *Bulleidia*, and *Acetobacter*, suggesting they are novel species within these known genera. In particular, two MAGs (*JAEXAI01* and *JAUZPN01*) lacked close reference genomes and were assigned new placeholders at the genus level, indicating that they may represent novel genera (Table 1). MMonitor also annotated the MAGs and mapped the annotations to the Kyoto Encyclopedia of Genes and Genomes (KEGG) database to create metabolic maps that show the metabolic potential of a MAG.

**Table 1:** High-quality MAGs recovered from nanopore WGS

MAG ID	Completeness (%)	Contamination (%)	Genome size (bp)	GTDB classification	Closest reference genome (ANI %)
bin.2	94.63	0.13	801,950	*Pseudoclavibacter_A caeni*	GCF_008831125.1 (99.28%)
bin.5	95.15	1.04	2,551,952	*Methanobacterium_C congolense*	GCF_900095295.1 (97.88%)
bin.6	95.58	0.05	6,495,533	*Dysosmobacter* sp.	no close reference
bin.11	96.77	0.53	2,816,210	*Bulleidia* sp.	no close reference
bin.12	91.25	0.30	1,765,253	*JAEXAI01* sp.	no close reference (putative novel lineage)
bin.13	99.96	1.05	3,110,456	*Acetobacter* sp.	no close reference
bin.17	91.03	2.30	4,688,061	*JAUZPN01* sp.	no close reference (putative novel lineage)

MAGs meeting MIMAG “high-quality” criteria (completeness ≥90%, contamination <5%), with GTDB taxonomic annotation and the closest reference genome (ANI, %).

**Figure 4:**
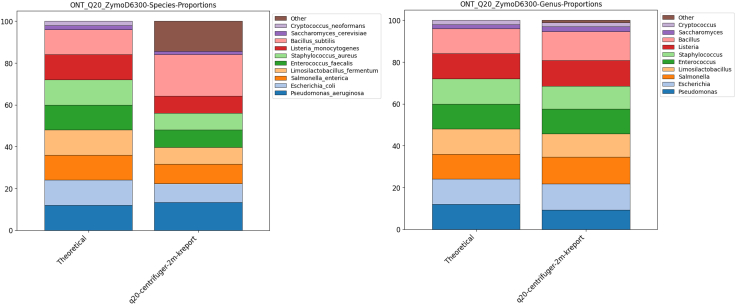
Taxonomic classification accuracy of MMonitor on the Zymo Q20 ONT mock community Stacked bar plots show the relative abundances of the most abundant taxa compared to the theoretical composition of the dataset. (A) Species-level classification. (B) Genus-level classification. The category “Other” includes low-abundance taxa and unclassified reads below the top 10 most abundant groups.

Finally, to benchmark the performance of MMonitor’s WGS pipeline for taxonomic abundance estimation, we analyzed the ONT Q20 Zymo WGS mock community, following the preprocessing described in Portik et al.^39^ Figure 4 shows MMonitor-estimated abundances versus the theoretical composition, generated with the Python notebook provided in Portik et al.^39^

### Modified versions of popular algorithms for more efficient real-time monitoring

To optimize MMonitor for real-time tracking, we have made minor adjustments to taxonomic profilers without affecting result quality. Emu now optionally saves reference indices on the hard drive, avoiding recomputation during sequencing runs and improving runtime, especially with SSDs. For high-performance systems, users can increase the batch size when Emu uses minimap2, reducing input/output operations at the cost of more memory use. Centrifuger is modified to hold indices in memory and process new sequences immediately, lowering runtime for small batches where index loading is a bottleneck, enhancing efficiency in real-time scenarios. These changes do not alter the behavior of the core algorithm and can be disabled if necessary. We also introduced updated reference indices that can be downloaded from MMonitor for users who need current databases, along with an option to retrieve sequences and rebuild the indices of chosen domains.

### Feature comparison with similar software

To position MMonitor within the landscape of other metagenomics tools, we compared its features to those of existing software. The primary focus of this comparison was on tools that support real-time data processing, including minoTour,^22^ Nanometa Live,^25^ MARTi,^24^ NanopoReaTA,^23^ EPI2ME,^32^ boardION,^33^ and CRuMPIT^34^ (see Table 2). We examined these tools according to different criteria that influence the usability of a software for real-time monitoring of metagenomes.

**Table 2:** Feature comparison of real-time metagenome tools

Feature	MMonitor	minoTour	Nano poReaTA	MARTi	Nanometa Live	MAIRA	EPI2ME	boardION	CRuMPIT
Time-series visualization	yes	yes	yes	yes	no	no	no	yes	no
Sample management	yes	yes	no	partly	no	partly	partly	no	no
Processing speed	fast	fast	fast	mod	fast	fast	fast	mod	mod
Sequence quality control	yes	yes	yes	yes	yes	partly	yes	yes	yes
Statistical analysis	yes	yes	partly	yes	partly	partly	partly	partly	no
Assembly support	yes	no	no	no	no	no	no	no	no
User interface	web, GUI, CLI	web	web, CLI	GUI	GUI	GUI, CLI	GUI	GUI	CLI
Data accessibility	web	web	no	web	no	no	web	no	no
Customizable databases	yes	yes	yes	yes	yes	yes	yes	no	yes
Automated reporting	yes	yes	partly	partly	partly	yes	yes	no	no
Scalability	high	high	mod	high	mod	high	high	mod	high
Open source	yes	yes	yes	yes	yes	yes	partly	yes	yes
Cost	free	free	free	free	free	free	free	free	free
Ease of installation	easy	mod	easy	easy	easy	easy	easy	mod	mod
Real-time analysis	yes	yes	yes	yes	yes	yes	yes	yes	yes

Nine tools compared across 15 criteria. Yes, fully met; partly, partially met; no, not met; mod, moderate.

“No” under data accessibility indicates local-only use; “web” denotes remote access.

### Detailed feature comparison

All tools in Table 2 support real-time processing of nanopore data, but they differ in algorithms, downstream analyses, and usability. Several tools (e.g., minoTour, MARTi, NanopoReaTA, Nanometa Live) provide real-time taxonomic profiling but lack *de novo* metagenome assembly. Of the compared tools, only MMonitor implements a complete metagenome assembly pipeline with the ability to generate MAGs from long-read data alone. EPI2ME includes an assembly workflow for single isolates, but not for community metagenomes.

#### minoTour

minoTour is a web-based, Django-backed LIMS for ONT devices that provides real-time metrics, run tracking, and taxonomic profiling during sequencing.^22^ It integrates adaptive sequencing workflows (e.g., ARTIC) and can be self-hosted. minoTour does not implement downstream metagenomic analyses (assembly, diversity/correlation time series) and requires users to deploy their own instance, which may limit accessibility for non-technical users.

#### NanopoReaTA

NanopoReaTA is a real-time analysis toolbox focused on RNA/cDNA data (differential transcriptomics) from ONT.^23^ It is distributed via Docker for portability; command-line operation and Linux requirement can be barriers. It is useful for transcription monitoring in real time, but it does not target multi-time point microbiome tracking or metagenome assembly.

#### MARTi

MARTi is an open-source, browser-based platform for real-time analysis and visualization of nanopore metagenomic data.^24^ It provides dynamic community composition and AMR gene reporting with documented installation and a public demo. It is well suited to rapid clinical or research use and does not focus on long-term time-series management or correlation/diversity dashboards. Currently, MARTi lacks support for metagenome assembly and full-genome functional metagenomics, as it is limited to reporting AMR and taxonomic profiles. Its utility strongly depends on up-to-date reference databases, but it does not provide automatic database updates. Users must rely on the developers to periodically create and release new versions.

#### Nanometa Live

Nanometa Live is a user-friendly application for real-time metagenomic analysis and pathogen identification that streams ONT reads into taxonomic profiling (Kraken2) with a GUI.^25^ It can run offline once installed. It does not include genome assembly/annotation workflows or time-series/diversity/correlation dashboards; it focuses on immediate species identification and run-time visualization.

#### MAIRA

MAIRA is a standalone, Java-based program for interactive taxonomic and functional analysis of long-read metagenomes on a laptop.^31^ It performs fast, online genus-level analysis with on-demand species-level and functional screens (e.g., AMR/virulence). It runs locally (no web access) and targets per-run real-time use rather than multi-time point tracking or dashboards.

#### EPI2ME

EPI2ME is a collection of ONT-supported workflows (EPI2ME Labs) for taxonomic profiling of amplicons and metagenomes, AMR screening, and more runnable on desktop or cloud.^32^ An isolate-genome assembly mode is available, but community metagenome assembly is not the target.^32^ Workflows are open source, whereas the platform itself is not; multi-sample time-series dashboards are not provided (advanced analyses typically require exporting results).

#### boardION

boardION is an interactive web application for real-time evaluation of ONT sequencing runs.^33^ It monitors instrument performance and run QC; it does not perform taxonomic profiling, assembly, or community analyses.

#### CRuMPIT

CRuMPIT is a real-time analytical pathway for clinical metagenomics (pathogen detection) using nanopore data.^34^ It emphasizes rapid identification rather than community-ecology features (e.g., time-series dashboards, diversity/correlation analyses).

Taken together, these comparisons highlight missing features across existing tools (e.g., time-series dashboards, sample management, configurable databases, and assembly for long reads). To address these limitations, we developed MMonitor.

#### MMonitor

Uniquely among the tools compared, MMonitor integrates full metagenome-assembly pipelines that generate high-quality MAGs and perform annotation-based functional analysis, enabling users to link taxonomic and functional profiles directly from long-read data. It is also the only tool that supports the automatic retrieval and updating of reference databases, ensuring analyses always use current data without manual intervention. Beyond these capabilities, MMonitor is designed for accessibility: it ships as prebuilt binaries with minimal setup, is fully open source, and provides both a GUI and CLI with support for remote computation. Samples are organized for easy comparisons, and custom visualizations (e.g., horizon plots) help reveal temporal trends. Pipelines scale efficiently to large datasets, and the dashboard supports interactive exploration across many samples, including very large datasets through filtering and subsetting options. The software runs on multiple platforms with bundled dependencies, includes a Docker container for easy dashboard deployment, and offers an offline mode for secure local inspection of results.

### Feature comparison with less direct competitors to MMonitor

Table 3 compares MMonitor with indirect competitors that emphasize broad metagenome analysis but lack real-time monitoring. Tools such as MG-RAST, MEGAN, and Anvi’o provide powerful statistical and functional analyses for large studies, yet are not suited to time-sensitive workflows.

**Table 3:** Comparison of MMonitor with indirect competitors that emphasize broad metagenome analysis rather than real-time monitoring

Feature	MMonitor	MG-RAST	MEGAN	Anvi’o	QIIME 2	MetaPhlAn 4	Nephele	PATRIC	MGnify
Time-series visualization	yes	limited	limited	limited	yes	limited	limited	yes	limited
Sample management	yes	limited	no	yes	limited	no	limited	yes	yes
Speed	fast	moderate	moderate	variable	variable	fast	moderate	moderate	moderate
Sequence quality control	yes	yes	yes	yes	yes	no	yes	yes	yes
Statistical analysis methods	yes	limited	yes	yes	yes	limited	yes	yes	yes
User interface	GUI & CLI	web-based	GUI & CLI	GUI & CLI	CLI	CLI	web-based	web-based & CLI	web-based
Data accessibility	web, API	web	server	no	no	no	limited	web	web
Customizable databases	yes	no	yes	yes	yes	limited	limited	yes	limited
Automated reporting	yes	yes	limited	limited	no	limited	yes	yes	yes
Scalability	high	moderate	moderate	variable	variable	high	high	high	high
Open source	yes	yes	yes	yes	yes	yes	yes	yes	yes
Cost	free	free	free (academic)	free	free	free	free	free	free
Ease of installation	easy	N/A	easy	moderate	moderate	easy	easy	easy to moderate	easy
Real-time analysis	yes	no	no	no	no	no	no	no	no

Cells indicate support level (yes, limited, or no). Abbreviations: GUI, graphical user interface; CLI, command-line interface; N/A, not applicable.

MG-RAST is an online platform for automated phylogenetic and functional analysis of metagenomic data, tailored to short-read datasets analyzed after server upload. It generates standard visualizations (e.g., bar charts for taxonomy) alongside advanced views such as principal coordinate analysis (PCoA), rarefaction, and KEGG maps, and provides metrics like richness and evenness. Maintenance status and reference updates can affect detection performance. In our hands, short-read data from a reactor metagenome yielded genus-level assignments only, limiting its utility for precise monitoring. Although MG-RAST includes sample management, it lacks efficient multisample comparison and time-series analysis. As a web service optimized for short reads, it is unsuitable for real-time monitoring and requires data uploads with typical queue times.

MEGAN^40^ is a desktop application with a GUI for taxonomic and functional analysis. It implements numerous algorithms and visualizations and supports multi-sample comparisons. However, it is not optimized for real-time usage or time-series visualization. MEGAN typically requires prior alignment against a protein reference database, which is efficient for large batches but less suitable for frequent, small increments common in monitoring.

Anvi’o^41^ is an open-source platform for metagenomic analysis, including binning, pangenomics, and visualization. It supports customizable databases and advanced statistics but uses a command-line interface with a steep learning curve and is not tailored for real-time monitoring or nanopore data.

QIIME 2 is an extensible microbiome analysis package focused on interactive analysis and visualization. It provides pipelines for taxonomic classification and diversity analyses with a plugin architecture but is primarily designed for amplicon (16S rRNA gene) data and is not optimized for long-read or real-time sequencing; the command-line interface may limit accessibility.

Nephele^42^ is a cloud-based microbiome platform with user-friendly web interfaces integrating tools such as QIIME, mothur, bioBakery, and a5-miseq. It supports amplicon and shotgun metagenomes and leverages AWS for scalable compute, reducing local burden. Reproducibility is emphasized by tracking inputs, parameters, and VM images.

PATRIC^43^ is a comprehensive bacterial bioinformatics resource focused on human pathogens. It integrates genomes using RAST and provides comparative genomics tools (genome browsing, protein family sorting, and pathways) plus community-derived data (disease information, experiments, and literature) for infectious disease research.

MGnify^44^ (formerly EBI Metagenomics) provides assembly, analysis, and storage for microbiome sequences. It offers taxonomic and functional annotations across diverse datasets and supports metagenomic and metatranscriptomic data, including long reads. Standardized and versioned pipelines ensure consistent, reproducible analyses.

### Taxonomic classification of WGS MOCK dataset

MMonitor’s pipelines employ previously described methods that have been benchmarked by their authors; therefore, we did not perform extensive re-benchmarking here. Because MMonitor uses updated indices for taxonomic classification, we evaluated it on the ONT Q20 Zymo WGS mock community, following the preprocessing described in Portik et al.^39^ Figure 4 shows MMonitor-estimated abundances versus the theoretical composition, generated with the Python notebook provided by Portik et al.^39^

MMonitor identified all genera, with only slight deviations from theoretical abundances. At the species level, MMonitor correctly classified the bacterial genomes but did not assign reads to *Cryptococcus neoformans* (yeast); instead, reads were assigned to a closely related *Cryptococcus* species. Species-level abundances were less accurate than genus-level abundances due to additional false-positives summarized as “Other” (Figure 4). Overall, MMonitor captured the taxonomic composition of the mock community and aligned closely with theoretical expectations.

## Discussion

Recent advances in sequencing technologies, along with their increasing application in clinical, environmental, and industrial microbiology, highlight the need for accessible and optimized tools for metagenome monitoring. However, the costs of sequencing and downstream analysis remain a practical limitation. These expenses vary depending on the sequencing platform, library preparation strategy, and computational setup and include not only sequencing itself but also consumables, reagents, and computational infrastructure. Nanopore sequencing, although generally more expensive than short-read approaches, enables species- and strain-level resolution as well as real-time analysis, but at the cost of higher error rates. Real-time local analysis reduces infrastructure needs and allows on-site sequencing, but consumables such as flow cells, reagents, and computational capacity must also be considered. Alternatives to WGS-based monitoring include targeted molecular assays, such as 16S rRNA gene profiling with meta-barcoding or hybridization probe capture. These approaches can quantify specific microbes with high sensitivity; however, they are restricted to targets with high similarity and may not capture the overall composition of the community.

### Possible applications for monitoring

MMonitor has a diverse range of applications, not limited to certain environments or species. It can be applied to any nanopore sequencing data, from metagenomes or isolates, and supports both WGS and targeted sequencing. In environmental biotechnology, it can be used to monitor microbial populations in bioreactors, aiding optimization of production processes for biofuels or bioremediation.

Metagenomic monitoring can uncover novel functional genes and enzymes, including those from uncultivable microorganisms, which can be harnessed to improve biomass conversion and biofuel production.^45^ It can also track shifts in microbial community composition and activity, providing information needed to optimize and control microbiome behavior for greater yield, stability, and a broader product range in large-scale biomanufacturing systems, as emphasized in Scarborough et al.^46^ In clinical microbiology, real-time monitoring of microbial communities can assist in tracking pathogen emergence and antibiotic resistance patterns. The ability to correlate microbial abundances with environmental or clinical metadata enhances the utility of MMonitor in these contexts.

Although metagenome monitoring has not yet been standardized as part of routine production workflows, several large-scale applications already demonstrate its feasibility. For example, shotgun metagenomics has been used to characterize resistomes in wastewater treatment plants,^47^ to profile microbial dynamics across sewer networks,^48^ and to monitor bioleaching microbial communities via high-throughput sequencing.^49^ These studies show that although sequencing turnaround currently limits fully real-time feedback, metagenome monitoring is already integrated into industrial workflows and is expected to expand as sequencing costs and analysis latency decrease.

### Relevance of reactor systems to this study

MMonitor was validated in two complementary contexts. BES reactors provided short, controlled enrichments with frequent 16S sampling, enabling rapid taxonomic tracking and diversity analyses. In contrast, AF and UASB reactors from a separate MCFA project supplied long-running WGS datasets to demonstrate genome assembly, binning, and functional monitoring.

Together, these systems highlight the dual-use cases of MMonitor: (1) rapid 16S-based tracking (BES) and (2) genome-resolved functional monitoring in continuous bioprocesses (AF/UASB). The BES itself was only indirectly related to MMonitor, but its time series offered a stringent test case for the 16S pipeline.

### Automation and predictive analytics

Another development avenue is the automation of sampling and library preparation. Manual collection, DNA extraction, and library construction remain time consuming and variable. Many wastewater facilities already use autosamplers as part of monitoring workflows,^50^ and open-source automation platforms such as Opentrons and VolTRAX are emerging to standardize metagenomic library preparation.^51^

In addition, MMonitor’s database structure provides opportunities for predictive analyses. By continuously storing and organizing metagenome data, the system could support downstream applications such as statistical modeling, machine learning, or other AI-based approaches to detect patterns and anticipate community dynamics. This would extend MMonitor beyond monitoring toward predictive microbiome analytics.

### Conclusion

In summary, MMonitor addresses current limitations in real-time metagenome analysis by providing a fully integrated solution that combines rapid taxonomic profiling, *de novo* assembly, and intuitive time-series visualization. By automating data analysis and key steps such as database updates and sample management, MMonitor enables users to capture dynamic shifts in microbial populations. Validation on bioreactor samples demonstrated its reliability for both 16S rRNA gene and WGS data, and the comparison with existing tools highlights complementary strengths across the field. As an open-source project, MMonitor welcomes contributions from the scientific community, fostering continuous improvement and broader adoption across microbiome research. Looking ahead, integration of automated sampling and preparation workflows could further enhance its utility by enabling near-continuous monitoring in industrial and clinical settings.

### Limitations of the study

Despite its strengths, MMonitor has certain constraints and areas for improvement.

• High sequencing loads: Very frequent updates (e.g., from multiple sequencers) may overburden consumer-grade hardware, as current optimizations target moderate analysis intervals.
• OS restrictions: Although the desktop app runs on multiple platforms, the underlying bioinformatics pipelines require a Unix-based environment. Full native support for Windows is not yet available.
• Short-read integration: MMonitor is optimized for nanopore data. While the WGS pipeline uses a genome database compatible with both long- and short-read data, short-read workflows have not been extensively tested. Future releases will include full short-read support (16S rRNA gene profiling and assembly). Other long-read technologies should also work but remain untested.
• Database coverage: Default references focus on bacteria, Archaea, and fungi. Viral or other specialized targets may require manual database creation and indexing.

Planned improvements include

• Containerization: Docker or Singularity support to simplify cross-platform usage, including Windows.
• Expanded functional analyses: Integration of antibiotic resistance gene monitoring and methylation detection (via Dorado) for pathogen surveillance and microbial regulation studies.
• Broader input support: Incorporation of transcriptomic, proteomic, or metabolomic data and compatibility with additional sequencing technologies.
• Performance optimization: GPU acceleration, improved I/O handling, and optimized reference databases for faster, large-scale analyses.

## Resource availability

### Lead contact

Further information and requests for resources should be directed to and will be fulfilled by the lead contact, Daniel H. Huson (daniel.huson@uni-tuebingen.de).

### Materials availability

This study did not generate new unique reagents.

### Data and code availability

• Raw 16S rRNA gene sequencing and WGS data have been deposited at NCBI under BioProject NCBI: PRJNA1216071 (https://www.ncbi.nlm.nih.gov/bioproject/PRJNA1216071). Reactor designs and cultivation protocols are described in detail in this study and referenced publications.
• The MMonitor source code (version 0.2.1), built binaries, and usage instructions are available on GitHub (https://github.com/lucast122/mmonitor) and archived on Zenodo under https://doi.org/10.5281/zenodo.17376966.
• Database-building scripts are included in the MMonitor repository. Required databases can be downloaded through the graphical user interface or referenced directly via the repository documentation.
• A hosted instance of the MMonitor dashboard is available at https://www.mmonitor.org.
• Any additional information required to re-analyze the data reported in this paper is available from the lead contact upon request.

## Acknowledgments

The authors thank the Angenent Lab and the Ley Lab for their sequencing data, suggestions, and tool testing. We also acknowledge members of the Algorithms in Bioinformatics lab at the University of Tübingen. Special thanks to Byoung Seung Jeon for building and sequencing the reactor that provided the WGS data. The development of MMonitor was funded through the Deutsche Forschungsgemeinschaft under Germany’s Excellence Strategy (EXC 2124–390838134) to R.E.L., L.T.A., and D.H.H. Additional parts of the work were funded by the 10.13039/100005156Alexander von Humboldt Foundation in the framework of the Alexander von Humboldt Professorship to L.T.A. and by the 10.13039/501100009708Novo Nordisk Foundation CO2 Research Center (CORC) with grant number NNF21SA0072700 to L.T.A. A.G. used the de.NBI Cloud, provided by the 10.13039/501100018929German Network for Bioinformatics Infrastructure (de.NBI) and ELIXIR-DE (Forschungszentrum Jülich; projects W-de.NBI-001, W-de.NBI-004, W-de.NBI-008, W-de.NBI-010, W-de.NBI-013, W-de.NBI-014, W-de.NBI-016, and W-de.NBI-022), and the High Performance and Cloud Computing Group at the Zentrum für Datenverarbeitung of the University of Tübingen, the state of Baden-Württemberg through bwHPC, and the German Research Foundation (DFG) for funding under “Project number 455787709” (bwForCluster BinAC 2).

## Author contributions

T.N.L. conceived the main scientific ideas, wrote most of the software, led data analysis and interpretation, and wrote the majority of the manuscript. U.B. generated most of the experimental data and contributed to analysis and writing. A.G. contributed to scientific ideas, analysis, and writing. K.G. assisted with data generation. T.L. and S.K. contributed to scientific ideas and the code. R.E.L. provided scientific input. L.T.A. contributed to scientific ideas, interpretation, and manuscript writing. D.H.H. contributed to scientific ideas, interpretation, and manuscript writing and acted as corresponding author. All authors provided feedback and helped improve the manuscript.

## Declaration of interests

The authors declare no conflict of interest.

## Declaration of generative AI and AI-assisted technologies in the writing process

During the preparation of this work, ChatGPT-5 (OpenAI) was used to rephrase text, identify typographical and formatting errors, and assist in literature searches. Portions of the Django server (API and request handling) and GUI (webpage html, css, and user configuration window) boilerplate code were generated with the assistance of Claude Sonnet 3.7 (Anthropic) to speed up development of routine components. All content was carefully reviewed and edited by the author, who takes full responsibility for the final manuscript.

## STAR★Methods

### Key resources table

**Table:** 

REAGENT or RESOURCE	SOURCE	IDENTIFIER
**Biological samples**		

Human stool samples (BES inoculation)	This paper	N/A
Chain-elongating microbiome (AF/UASB inoculum)	This paper	N/A

**Chemicals, peptides, and recombinant proteins**		

16S Barcoding Kit 1–24 (SQK-16S024)	Oxford Nanopore Technologies	Cat# SQK-16S024
LongAmp Hot Start Taq 2x Master Mix	New England Biolabs	Cat# M0287
AllPrep® PowerFecal Pro DNA/RNA Kit	QIAGEN	Cat# 80244
Qubit™ Flex Fluorometer	Invitrogen	Cat# Q33226
R9.4.1 Flow Cell (FLO-MIN106)	Oxford Nanopore Technologies	Cat# FLO-MIN106
NanoPhotometer™ N60/N50	Implen	Cat# N60/N50

**Deposited data**		

Raw 16S and WGS data	NCBI BioProject	NCBI: PRJNA1216071
Annotated MAGs, annotations, KEGG maps	GitHub	https://github.com/lucast122/mmonitor
Hosted MMonitor dashboard instance	This paper	https://www.mmonitor.org

**Software and algorithms**		

MMonitor (desktop & dashboard)	This paper	https://github.com/lucast122/mmonitor
Centrifuger	Mourisl et al.	https://github.com/mourisl/centrifuger
Emu (16S profiler)	Curry et al.^12^	https://github.com/treangenlab/emu
Minimap2 v2.26	Li^52^	https://github.com/lh3/minimap2
Flye v2.9	Kolmogorov et al.^53^	https://github.com/fenderglass/Flye
Medaka v2.0.1	Oxford Nanopore Technologies	https://github.com/nanoporetech/medaka
Metabat2 v2.15	Kang et al.^54^	https://bitbucket.org/berkeleylab/metabat/src/master/
GTDB-Tk v2.4.0	Chaumeil et al.^55^	https://github.com/Ecogenomics/GTDBTk
CheckM2	Chklovski et al.^38^	https://github.com/chklovski/CheckM2
Bakta v1.9.4	Schwengers et al.^56^	https://github.com/oschwengers/bakta
KEGGCharter v1.0.2	Sequeira et al.^57^	https://github.com/iquasere/KEGGCharter
Biopython seqIO v1.83	Cock et al.^58^	https://biopython.org
Scikit-bio v0.5.9	scikit-bio developers	http://scikit-bio.org
Django v5.0	Django Software Foundation	https://www.djangoproject.com
Plotly Dash v2.16	Plotly	https://dash.plotly.com
Docker	Docker Inc.	https://www.docker.com

### Experimental model and subject details

#### Microbial samples

Two distinct reactor systems were studied. For 16S rRNA gene sequencing experiments, three bioelectrochemical system (BES) reactors were inoculated with fresh human fecal samples collected with informed consent and processed anaerobically (see Method details). For WGS experiments, two continuous bioreactors (anaerobic filter and UASB) were inoculated with a chain-elongating microbiome derived from a previously operated CSTR and maintained over a period of ∼ 1000 days. This experiment was approved by the Ethics Committee of the Medical Faculty of the University of Tübingen (Project number: 456/2023A). Anonymized stool samples were collected with the permission of all human subjects.

### Method details

#### Analysis pipeline

MMonitor was written in Python (v3.11). The desktop application manages data input and pipeline execution, while the computations are performed by external bioinformatics tools (Figure 1B). Benchmarks and methods for these tools are available in their respective publications.

All input reads are first processed by Filtlong (v0.2.1) for quality filtering. Parameters can be set in the Analysis Configuration tab of MMonitor Desktop. By default, WGS reads shorter than 2000 bp and 16S rRNA gene reads shorter than 1000 bp or longer than 2000 bp are discarded, as well as reads with PHRED scores below 10. Only reads that pass this filtering are used for downstream analysis. Basic quality statistics (quality scores, read lengths, and base counts) are computed directly from input reads using Biopython SeqIO.^58^

For taxonomic analysis of nanopore WGS data, MMonitor uses Centrifuger,^59^ which applies the Burrows–Wheeler transform (BWT) and Ferragina–Manzini (FM) index.^60^^,^^61^ Centrifuger is similar to Centrifuge,^13^ but employs run-block compression to reduce memory requirements with only a modest increase in runtime. For nanopore 16S rRNA gene profiling, MMonitor relies on Emu,^12^ which combines minimap2^59^ alignments with an expectation–maximization algorithm^62^ to refine relative abundances.

For functional analysis, WGS reads are required. The assembly pipeline follows previous work.^63^ Reads are assembled into contigs using Flye^53^ with the --meta flag and, by default, --nano-raw. Users can specify --nano-hq if basecalling was performed with a sup model. The contigs are polished using Medaka (v2.0.1).^64^ Medaka attempts to auto-detect the correct model, but if data were basecalled with an old model or are not in pod5 format, users must supply the correct model manually (see documentation: https://github.com/nanoporetech/medaka).

The consensus assembly is binned with MetaBAT2,^54^ and resulting bins are annotated taxonomically using GTDB-tk (v2.4.0).^55^ MAG quality is assessed with CheckM2^38^; bins with completeness above 80% and contamination below 10% are retained. Bakta (v1.9.4)^56^ is then used for functional annotation, which is mapped to KEGG pathways using keggcharter (v1.0.2).^57^^,^^65^

In addition to the GUI, MMonitor can be run in command-line mode, enabling remote execution on other systems (e.g., via SSH), as described in the project README.

#### Webserver and Dashboard

The web server backend was designed with the Django framework^66^ and the dashboard was implemented using a combination of Plotly Dash and JavaScript.^67^ Docker was used to bundle the dependencies of the server and can be used to deploy it. We offer a default online version of the MMonitor server that all users can access. However, if you set up your own MMonitor server, you gain full control over the database and user management, allowing you to customize access and data handling according to your needs.

In addition to visualizations, the dashboard also provides several statistical methods. Normalized counts $c_{n}$ (1) are calculated from raw counts $c_{r}$, the number of aligned bases *b*, and a scaling factor *f*:

(Equation 1) $$c_{n}=\frac{c_{r}}{b}\times f$$

The scaling factor is the average number of aligned bases across all samples and ensures that raw and normalized counts have a comparable magnitude.

Alpha and beta diversity are calculated using scikit-bio (v0.5.9) based on normalized counts. For alpha diversity, the Shannon (2) and Simpson (3) indices are used, where *S* is the number of unique taxa and $p_{i}$ is the relative abundance of taxon *i*:

(Equation 2) $$H=-\sum_{i=1}^{S}p_{i}\;\log_{2}p_{i}$$

(Equation 3) $$D=1-\sum_{i=1}^{S}p_{i}^{2}$$

Beta diversity is assessed using the Bray–Curtis distance (4) between all samples, where $u_{i}$ and $v_{i}$ are the proportions of taxon *i* in samples *u* and *v*:

(Equation 4) $$d\left(u,v\right)=\frac{\sum_{i}\left|u_{i}-v_{i}\right|}{\sum_{i}\left|u_{i}+v_{i}\right|}$$

PCoA is performed on Bray–Curtis distances using scikit-bio ordination functions. Horizon plots were implemented with the JavaScript D3 plotting library for each taxon, displaying differences in abundance relative to the previous sample.

Taxonomy–metadata correlations are computed with pandas and scikit-bio using Pearson (5), Kendall (6), or Spearman (7) correlation coefficients. Here, cov is the covariance, *X* is a series of metadata values, *Y* is the series of taxon counts, *C* and *D* are the numbers of concordant and discordant pairs, and R(X) is the rank of variable *X*.^68^^,^^69^

(Equation 5) $$\rho \left(X,Y\right)=\frac{\text{cov}\left(X,Y\right)}{\sigma_{X}\;\sigma_{Y}}$$

(Equation 6) $$\tau =\frac{C-D}{C+D}$$

(Equation 7) $$r_{s}=\rho_{R\left(X\right),R\left(Y\right)}=\frac{\text{cov}\left(R\left(X\right),R\left(Y\right)\right)}{\sigma_{R\left(X\right)}\;\sigma_{R\left(Y\right)}}$$

#### Reference database creation

MMonitor utilizes customized databases for taxonomic classification. The Database Manager window can be used to change profiler indices or to download and build fresh versions on demand. To save build time for users, we provide pre-built indices online (see data availability section). To create the Centrifuger index, we downloaded all complete genomes of bacteria, archaea, and fungi from the NCBI RefSeq database^70^ (as of October 2024) and concatenated them into a single file. We also downloaded the taxonomy tree and taxonomy mapping file from the NCBI taxonomy dump. The centrifuger index was then built using the centrifuger-build command with default parameters, providing the concatenated genomes, taxonomy tree, and mapping file previously downloaded.

For the Emu database we followed the authors’ in their methods section Emu 16S database. All 16S rRNA gene sequences for bacteria and archaea from the NCBI targeted loci directory and the rrnDB-5.9 sequences were concatenated into a single file. Then we downloaded the NCBI taxdump and accession-to-taxid mapping file and the mapping file for rrnDB-5.9. We used Emu’s build-database and Centrifuger’s internal script for building the databases. We did not include the index files in the MMonitor binaries due to their large file size and instead uploaded them to a public repository. If no index is found or provided by the user during run-time, MMonitor will notify the user and try to download it. The scripts for downloading and building the new databases are included in MMonitor and can be run on demand from the Database Manager of the desktop app. You can find the source code and custom index files in the Data Availability section.

While the 16S rRNA and WGS pipelines in MMonitor use different tools and databases, the WGS database is built from complete reference genomes (by default all RefSeq bacterial, archaeal, and fungal genomes). This design enables detection of any marker genes present in these genomes, making the same database creation scripts applicable to marker gene sequencing data, provided the target genes are part of the reference genomes. In future versions, we plan to add an option for users to supply a FASTA file with specific marker gene sequences, allowing the creation of dedicated marker-gene databases for faster querying and targeted monitoring.

#### 16S rRNA gene nanopore sequencing

For 16S rRNA gene amplicon sequencing, we used the 16S rRNA gene Barcoding Kit 1–24 (SQK-16S024, Oxford Nanopore Technologies) and an R9.4.1 flow cell (FLO-MIN106). The 16S rRNA gene Barcoding Kit enables rapid and full-length 16S rRNA gene sequencing for organism identification by using universal primers 27F (5′–AGAGTTTGATCMTGGCTCAG–3′) and 1492R (5′–CGGTTACCTTGTTACGACTT–3′).

For 16S rRNA gene amplification via PCR, we mixed 10 μL of 10 ng DNA with 25 μL of LongAmp Hot Start Taq 2× Master Mix (New England Biolabs), 10 μL of an individual 16S rRNA gene barcode, and 5 μL of nuclease-free water. PCR cycles were: 1 min at 95°C; 25 cycles of 20 s at 95°C, 30 s at 51°C, 2 min at 65°C; and a final elongation of 5 min at 65°C.

Afterward, bead cleaning, preparation of the cleaned-up DNA library with rapid adapter and pooling, and loading of the R9.4.1 flow cell were performed following the manufacturer’s protocol. The sequencing time depended on the number of barcodes pooled in one library. We decided to sequence for 2 h per barcode, resulting in reasonable coverage of the 16S rRNA gene for improved classification results. MinKNOW software version 23.11.4 was used for data generation.

#### Bioelectrochemical system setup (16S rRNA gene sequencing experiments)

The BES consisted of two glass chambers (working and counter) separated by a Nafion 117 ion-exchange membrane (Sigma), ensuring that the human fecal sample contacted only the working electrode ($E_{we}$). A three-electrode configuration ($E_{we}$, $E_{ce}$, $E_{ref}$) was used, with the working electrode poised at +350 mV vs. Ag/AgCl for H_2_ removal by oxidation. Both $E_{we}$ and $E_{ce}$ were carbon cloth electrodes (area: 122 cm^2^). $E_{we}$ was spray-coated on both sides with platinated carbon (10%) at a loading of 4 mg cm^−2^. The reference electrode ($E_{ref}$) was Ag/AgCl (silver wire chloridized, embedded in 3 M KCl and 0.7 g agarose).

Prior to assembly, Nafion 117 membranes were activated in 1 M sulfuric acid for 24 h and equilibrated for 24 h in a salt solution (85.5 mM NaCl, 16.9 mM Na_2_ HPO_4_ 2H^2^ O, 41.9 mM NaHCO_2_). Reactors (including the membrane-separated electrodes) were assembled in a sandwich configuration and sterilized at 121°C for 20 min under aerobic conditions. After autoclaving, chambers were filled with anaerobic, reduced medium and sparged with N_2_:CO_2_ prior to inoculation. The working volume was 21 mL in both chambers. The geometry minimized the microbe–electrode distance to ∼ 1 mm.

#### DNA extraction for BES (16S)

For DNA extraction, 500 μL of BES liquid was centrifuged (19,000 ×g, 5 min, 4°C) to pellet the cells. DNA was isolated with the AllPrep PowerFecal Pro DNA/RNA Kit (QIAGEN) using a FastPrep-24 5G bead-beater (2 cycles at 6.0 m s^−1^ for 40 s with 30 s rest). We followed the manufacturer’s instructions with one exception: <450 μL (instead of 300 μL) supernatant was taken in pretreatment step 6 (after adding CD2), following the vendor’s guidance for higher volumes. DNA quantity was measured with the Qubit Flex Fluorometer (Invitrogen) and DNA quality with the NanoPhotometer N60/N50 (Implen).

#### Continuous bioreactors with pertraction system (WGS experiments)

Two independently operated, continuously fed upflow bioreactors with integrated liquid–liquid extraction (pertraction) were run in parallel for ∼ 1019 days. Each bioreactor was a double-walled glass column (height 125 cm) maintained at 30°C and pH 5.5. One vessel contained wheel-shaped plastic packing (anaerobic filter, AF reactor); the second was an upflow anaerobic sludge blanket (UASB) reactor without packing to promote granule formation. Fermentation broth was recirculated through two hollow-fiber membrane contactors: medium-chain carboxylates were extracted into a hydrophobic solvent and subsequently transferred into an alkaline extraction solution. An in-line 0.8 L filter module protected the membranes during early operation.

##### Medium and inoculum

Both reactors were inoculated with 2 mL glycerol stock (0.04% v/v) from a chain-elongating microbiome previously maintained in a CSTR on ethanol/acetate feed. The defined medium contained ethanol (13.42 g L^−1^; ∼ 600 mM C) and acetate (3.12 g L^−1^; ∼ 100 mM C) at a 6:1 molar ratio, plus bicarbonate and nutrients.

##### Operating periods

Over 1019 days, four operating periods (I–IV) were defined by construction and oxygen-management changes: limiting oxygen intrusion (airlocks, N_2_ sparging, metal tubing), adjusting reducing agents (L-cysteine, sodium sulfide), and later introducing controlled O_2_ supply. Gas additions included N_2_/H_2_ (95/5% v/v; 0.3 ± 0.1 mL h^−1^) and air (11.8–17.1 mL h^−1^) during late operation. These interventions altered the redox balance and *n*-caprylate productivity.

##### Sampling

Biomass for metagenomics was collected from reactor columns and the filter module at Days 9, 283, 397/455, and 701.

### Quantification and statistical analysis

All statistical procedures, including normalized counts, alpha and beta diversity, and taxonomy–metadata associations, are implemented in the MMonitor dashboard and described in detail in the Webserver and Dashboard section (Equations 1, 2, 3, 4, 5, 6, and 7).

### Additional resources

Installation instructions, database management, and remote execution guidelines are available in the MMonitor README at www.github.com/lucast122/mmonitor.
